# Cooperative ankle-exoskeleton control can reduce effort to recover balance after unexpected disturbances during walking

**DOI:** 10.1186/s12984-022-01000-y

**Published:** 2022-02-17

**Authors:** Cristina Bayón, Arvid Q. L. Keemink, Michelle van Mierlo, Wolfgang Rampeltshammer, Herman van der Kooij, Edwin H. F. van Asseldonk

**Affiliations:** 1 Department of Biomechanical Engineering, University of Twente, Enschede, The Netherlands; 2 Department of BioMechanical Engineering, Delft University of Technology, Delft, The Netherlands

**Keywords:** Balance, Control, Exoskeleton, Ankle, Human–robot interaction, Gait

## Abstract

**Background:**

In the last two decades, lower-limb exoskeletons have been developed to assist human standing and locomotion. One of the ongoing challenges is the cooperation between the exoskeleton balance support and the wearer control. Here we present a cooperative ankle-exoskeleton control strategy to assist in balance recovery after unexpected disturbances during walking, which is inspired on human balance responses.

**Methods:**

We evaluated the novel controller in ten able-bodied participants wearing the ankle modules of the Symbitron exoskeleton. During walking, participants received unexpected forward pushes with different timing and magnitude at the pelvis level, while being supported (Exo-Assistance) or not (Exo-NoAssistance) by the robotic assistance provided by the controller. The effectiveness of the assistive strategy was assessed in terms of (1) controller performance (Detection Delay, Joint Angles, and Exerted Ankle Torques), (2) analysis of effort (integral of normalized Muscle Activity after perturbation onset); and (3) Analysis of center of mass *COM* kinematics (relative maximum *COM* Motion, Recovery Time and Margin of Stability) and spatio-temporal parameters (Step Length and Swing Time).

**Results:**

In general, the results show that when the controller was active, it was able to reduce participants’ effort while keeping similar ability to counteract and withstand the balance disturbances. Significant reductions were found for soleus and gastrocnemius medialis activity of the stance leg when comparing Exo-Assistance and Exo-NoAssistance walking conditions.

**Conclusions:**

The proposed controller was able to cooperate with the able-bodied participants in counteracting perturbations, contributing to the state-of-the-art of bio-inspired cooperative ankle exoskeleton controllers for supporting dynamic balance. In the future, this control strategy may be used in exoskeletons to support and improve balance control in users with motor disabilities.

**Supplementary Information:**

The online version contains supplementary material available at 10.1186/s12984-022-01000-y.

## Background

Wearable exoskeletons are powerful solutions that can be applied to reinforce and enhance mobility in able-bodied subjects [1, 2], or to restore lost functions of people with motor problems, such as those resulting from aging [3, 4], neurological disorders as spinal cord injury [5–7], or others [8–10]. Although these robotic devices are reliable in assisting individuals’ locomotion, researchers still struggle to design smart controllers for exoskeletons that also support balance when needed. Balance support is currently a serious demand and an often-heard wish of exoskeletons stakeholders, who consider this a fundamental and necessary skill [11, 12]. Especially during walking, balance becomes even more challenging, as recovery reactions to unexpected disturbances are often required to continue the gait cycle. During dynamic tasks, humans can exploit different balance recovery strategies, and the selected strategy may depend not only on the magnitude and direction of perturbation, but also on the perturbation timing within the gait cycle [13, 14]. Ideally, controllers for exoskeletons should be developed to take into account all these possible reactions.

One of the main issues of current lower-limb exoskeletons to achieve the challenge of balance is the insufficiency of human–robot interaction. This interaction is particularly significant when the prone-to-fall user still has some residual control. In these situations, *cooperative* controllers should be used to support in restoring balance only when necessary (e.g. onset of a potential fall). This may be known as “assist-when-needed” approach.

Recent studies with exoskeletons that developed “assist-when-needed” approaches to support balance were primarily focused on hip control [15–17]. The proposed controllers, provide hip torque to adjust the stepping location, either by supporting hip abduction-adduction (step-width adaptation) or hip flexion-extension (step-length adaptation). The assistance is triggered and modulated when perturbations are detected by using different feedback signals, such as the hip angle [15], the extrapolated center of mass (*XcoM*) [16], or the estimated leg force [17]. These approaches are not intended to replace human control, but rather to augment the user’s balance by providing the required assistance in synergy with the human wearer just after the onset of an imminent fall.

Although the hip joint is important for controlling the swing leg and preparing for foot placement, previous studies provided evidence that also the ankle joint during stance is crucial in balance maintenance [13, 14, 18]. The torque generated around the ankle acts to decrease the body’s velocity in the direction of the perturbation. Vlutters et al. [14] demonstrated that humans modulate the ankle joint torque of the stance leg as a response to antero-posterior (AP) pelvis perturbations. This ankle torque modulation scales with the provided perturbation magnitude, and thereby with subject’s center of mass (*COM*) kinematics after perturbation. Using the ankle strategy, subjects were able to eventually slow down the body movement provoked by the external disturbance.

Despite the demonstrated importance of the ankle joint, studies centered on ankle-exoskeleton controllers for assisting in balance during gait, and their effective evaluation with human users, are still scarce. Some preliminary approaches specifically designed for ankle balance support were mainly centered on stance situations. An example is the work presented in [19], in which the authors demonstrate that standing balance can effectively be supported by a strategy based on the user’s *COM* kinematics. Another example is the work of Ugurlu et al. [20], where the authors propose a real-time variable ankle stiffness as a balance control technique for standing with exoskeletons. Unfortunately this approach was not tested with the human wearer in the loop. Other methods that do use cooperative ankle-controllers during human locomotion did not address their effectiveness in counteracting balance recovery [21]. Finally, there have also been control approaches based on neuromuscular models that propose ankle balance assistance during walking with prosthetic legs [22]. Unfortunately, these models do not demonstrate the ability to generate cooperative human-like balance responses without specific supplementary additions [23].

In this work we have the aim of developing a “simple” bio-inspired control strategy for ankle-exoskeletons that works in synchrony with the human and effectively cooperates and assists balance recovery during walking. In our approach, we first detect disturbances to the *COM* in real-time by using human’s kinematics responses. Based on this detection, we trigger the robotic ankle assistance to recover stability. The assistance delivered by the controller tries to mimic humans ankle torque modulation [14], scaling with body kinematics and distributed proportionally over both ankles based on the weight supported by the corresponding leg. Our controller presents high levels of transparency during unperturbed locomotion [24], and provides appropriate support in synchrony with the human’s reaction, ensuring the “assist-when-needed” approach. A specific advantage of the proposed method is that it does not require specific subject personalization and thereby it can be easily applied without time-consuming tuning.

Our main hypothesis is that the developed ankle-exoskeleton controller is capable of reducing able-bodied users’ effort required to counteract unexpected perturbations during walking without detriment of their stability. Moreover, we expect the controller to be reliable in both, detecting the perturbations and providing assistance that works in sync with the user to eventually help in recovering balance.

## Methods

### Balance assistive controller

**Fig. 1:**
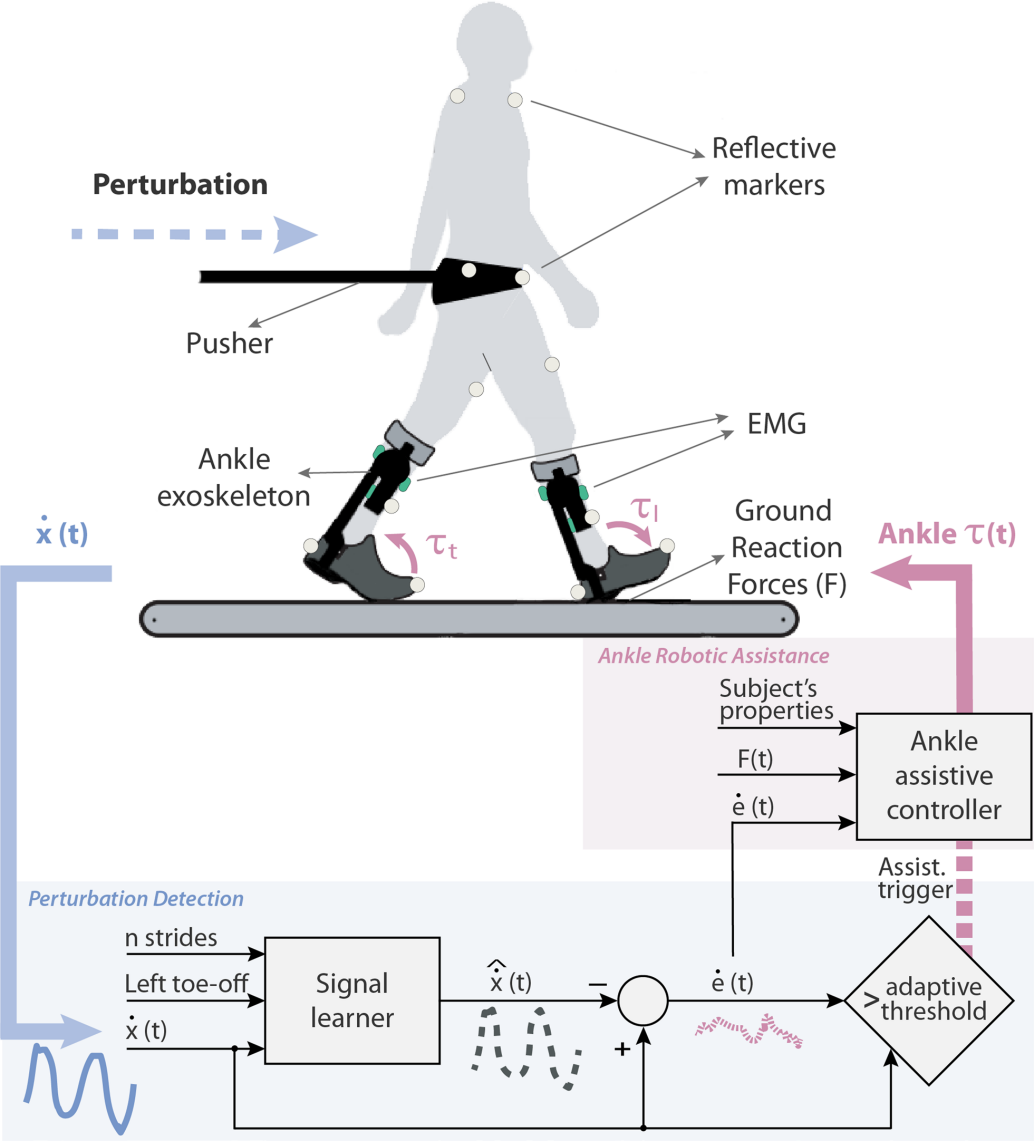
Controller overview. The real-time perturbation detection algorithm based on $\dot{COM}$ feedback (blue) triggers the robotic assistance controller (pink), which support balance by providing ankle plantar/dorsi-flexion torques

The developed controller is focused on providing ankle plantar/dorsi-flexion assistance to counteract perturbations in the sagittal plane (Fig. 1). The delivered assistance ($\tau$) is intended to support the user in balance recovery by accelerating their *COM* in the opposite direction of the provided perturbation. To achieve this goal, the controller was designed to detect unexpected perturbations by using the kinematic information of the user’s *COM* (loss of balance). Immediately (and only) after the detection of the onset of a perturbation, the controller triggers the robotic assistance. This assistance is delivered to the ankle(s) of the foot/feet that are in contact with the ground, i.e. to both ankles during double support phase and to the ankle of the stance leg during single support phase. For steps during unperturbed walking, the robotic assistance is set to zero and the exoskeleton is controlled to be transparent to the user [24], thus ensuring the “assist-when-needed” approach. The two main parts of the controller, (1) perturbation detection and (2) ankle robotic assistance, are presented in detail in the following subsections.

#### Perturbation detection

To detect a perturbation by means of the user’s *COM* information, we made the assumption that unperturbed walking can be considered as a quasi-periodic motor task [25]. Based on this assumption, the course of the *COM* during unperturbed locomotion can be predicted by using preceding strides. The onset of a postural transition due to an unexpected disturbance can be detected by a sudden and abrupt deviation of the actual kinematics from the predicted ones.

We developed a perturbation detection algorithm by means of three main components (blue area in Fig. 1): (1) signal learner, (2) *COM* kinematics divergence, and (3) adaptive threshold for triggering:

- The signal learner used $n = 5$ preceding strides to generate an (complete gait cycle) output signal corresponding to the estimated user’s *COM* velocity in the AP direction ($\hat{\dot{x}}$). The quasi-periodic input of the signal learner was the actual user’s $\dot{x}$, which was constantly fed back in real-time to the detection algorithm. The predicted learned output $\hat{\dot{x}}$ was calculated averaging the $\dot{x}$ input over the preceding $n = 5$ unperturbed interpolated gait cycles. The left toe-off (extracted from real-time ground reaction force data) was used to split the signal into gait cycles before being interpolated. Perturbed steps were excluded from calculating the learned output.
- For the *COM* kinematics divergence, the *COM* velocity error ($\dot{e}$) at every instant of gait was computed as:
  1 $$\dot{e} = \dot{x}- \hat{\dot{x}}$$
- Finally, we defined an adaptive threshold that used both the $\dot{x}$ and the $\dot{e}$ signals to detect perturbations and trigger the ankle-exoskeleton assistance accordingly. Instead of using a constant threshold, we considered the preceding $n = 5$ unperturbed gait cycles to adapt our perturbation detection. First, based on the results of pre-recorded data [26], we defined a hand-tuned constant factor $p = 1.5$ to detect the provided perturbations with the least false positive and false negative errors. Second, this factor was applied to the $\dot{x}$ and the $\dot{e}$ signals considering that a perturbation happened (and thereby the robotic assistance should be triggered) if any of the following statements were true:
  - In the current gait cycle *m* there is a maximum of $\dot{x}$ larger than *p* times the averaged maximum of $\dot{x}$ over the preceding *n* unperturbed gait cycles:
    2 $$\max \limits _{t}\left\{ ^m\dot{x}\left( t\right) \right\} > p \frac{1}{n} \sum _{i=m-n}^{m-1}\max \limits _{t}\left\{ ^i\dot{x}\left( t\right) \right\}$$
  - In the current gait cycle *m* there is a single measurement sample of $\dot{e}$ larger than *p* times the averaged $\dot{e}$ range over the preceding *n* unperturbed gait cycles:
    3 $$^m\dot{e} > p\frac{1}{n} \sum _{i=m-n}^{m-1}\left( \max \limits _{t}\left\{ ^i\dot{e}\left( t\right) \right\} - \min \limits _{t}\left\{ ^i\dot{e}\left( t\right) \right\} \right)$$
    where *t* in (2) and (3) is the local time value within duration of the gait cycle.
  Figure 2A shows an example of a real-time perturbation detection based on the previously described method.
  **Fig. 2:**
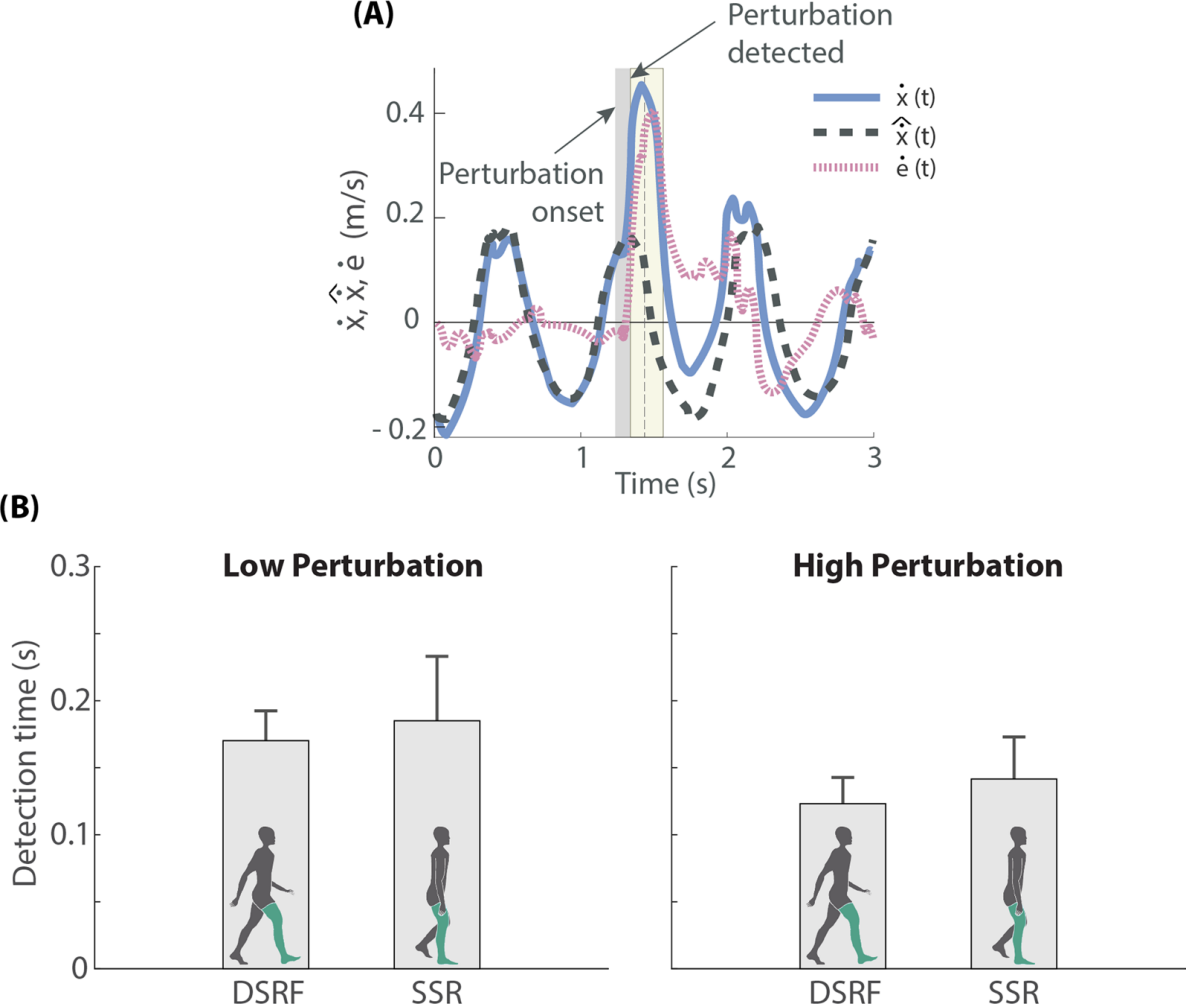
**A** Example of a real-time perturbation detection based on the detection algorithm described in *Perturbation detection* section. Vertical dark grey area represents the duration of the perturbation, and light yellow area the interval in which the robotic assistance was being triggered. **B** Detection times obtained during the experiment performed with 10 healthy subjects (mean ± SD), who received perturbations of two magnitudes (Low and High) at two moments of the gait cycle (*DSRF* double support with right leg in front, *SSR* single support right leg)

#### Ankle robotic assistance

The assistance applied to the ankles was designed to mimic the phenomenon of ankle torque modulation observed in healthy humans [14, 23]. To do that, we first made the robotic assistance, $\tau$ (Nm), proportional to $\dot{e}$ (m/s), thus adapting the support to the magnitude of the perturbation. Second, the assistance was scaled with the vertical ground reaction force, *F* (N), individually measured below each foot (feet), to make sure that the torques were properly distributed over each leg in contact with the ground. Finally, as the reactive response also depends on the user’s properties, the subject’s mass, *M* (kg), and the subject’s *COM* height, $l_{com}$ (m), were also taken into account to scale the assistance. According to this, the torque-based assistance was defined as displayed in (4). These torques were applied as long as conditions (2) OR (3) were satisfied.

4 $$\tau _{l/t} = K\; M\sqrt{g \; l_{com}}\ \dot{e} \frac{F_{l/t}}{F_{l}+F_{t}}$$

where the subscripts *l* and *t* refer to the leading and trailing legs respectively; and *K* is a dimensionless constant factor that was empirically tuned based on pre-recorded data [26]:

5 $$\left\{ \begin{array}{ll} K = +0.43\; \text {for}\; \text{ plantar-flexion}\; \text{torque} \\ K = -0.43\; \text {for}\; \text {dorsi-flexion}\; \text {torque} \end{array}\right.$$

To determine whether a leg should receive dorsi- or plantar-flexion support, the relative position of the *COM* with respect to the stance foot (feet) was checked. The main principle is to accelerate the *COM* in the opposite direction of the provided perturbation, thus helping to recover balance. With a forward perturbation, if the foot is in front of the *COM* (leading), ankle plantar-flexion assistance is required. That makes that the center of pressure moves to the toes, which would slow down the forward movement provoked by the perturbation. When the foot is behind the *COM* (trailing), a dorsi-flexion torque is applied to slow down heel lift and forward propulsion (third rocker of gait [27]). This same principle is applied during both, single and double stances, with the unique difference that during single stance, no assistance is applied to the leg that is in swing phase as the correspondent measured *F* is zero. The assistive torques would change direction with a backward perturbation as the result of a negative $\dot{e}$.

As mentioned, assistive torques were applied only during the detection of a perturbation. The rest of the time, the ankle-exoskeleton worked in transparent mode ($\tau _{l/t} = 0$). The changes in torque were rate limited with rising and falling rates of ± 250 Nm/s to prevent abrupt responses.

### Robotic exoskeleton

The Symbitron [5] is a force-controlled modular lower-limb exoskeleton with bilateral actuation on hip, knee and ankle. For this experiment, only the ankle modules of the exoskeleton were used. These ankle modules have four degrees of freedom, of which two are active and were used by the controller (bilateral plantar/dorsi-flexion), and two are passive (bilateral inversion–eversion).

### Experiment

Ten able-bodied participants (six male) took part in this study (weight 65.47 ± 7.01 kg, height 1.74 ± 0.09 m, age 28.1 ± 1.73 years-old). The experimental protocol was approved by the local ethical committee of the University of Twente. All participants gave written informed consent prior to the experiment.

The experiment consisted of a single session composed of six different perturbed walking trials of 5 min each (Table 1), where three walking conditions were tested: Exo-NoAssistance, Exo-Assistance and NoExo. During the trials, the participants were asked to walk on a dual-belt treadmill (Y-mill, Motek Medical, Amsterdam, The Netherlands) at a constant speed of 2 km/h, while they received external forward perturbations from a pusher device (Moog, Nieuw-Vennep, Netherlands) attached to their pelvis by a soft brace (Fig. 1). The pelvis was chosen as point of application of the external perturbation, as it approximately coincides with the height location of the whole-body *COM*. This avoids causing major body rotation and allowed the investigation of balance responses to changes in linear *COM* motion. Participants were instructed to continue walking as normal as possible after perturbations occurred, ensuring that their feet landed on the corresponding belt of the dual-belt treadmill.

**Table 1:** Trials performed during the experiment

W. Condition	Exo				NoExo	
	NoAssistance		Assistance			
Trial	1	2	3	4	5	6
P. Magnitude	Low	High	Low	High	Low	High
	10%	16%	10%	16%	10%	16%
	U + E	U + E	U + E	U + E	U	U
P. Occurrence	Semi-randomized at two moments of the gait cycle: DSRF and SSR					

Three Walking Conditions (Exo-NoAssistance, Exo-Assistance, NoExo) were assessed with two Perturbation Magnitudes (Low—10% weight, High—16% weight) each. U and E refer to the weight of the user and exoskeleton respectively. Perturbations occurred at DSRF and SSR moments during gait (Perturbation Occurrence). The order of the trials was quasi-randomized

Two magnitudes of perturbation (square force pulse of 0.2 s, forward direction) were applied with the external pusher: Low (10% of user + exoskeleton weight) and High (16% of user + exoskeleton weight). For trials without exoskeleton, only the user’s weight was considered in determining the perturbation magnitudes.

The onset of the perturbations happened at two different moments during the gait cycle (perturbation occurrence): DSRF—coinciding with the start of the double support phase with the right foot leading; and SSR—at mid single stance right (left foot in swing). The occurrence of DSRF or SSR perturbations within a trial were quasi-randomized. The time between perturbations was also randomized to prevent anticipation and varied between 6 and 10 s.

The order of the trials was quasi-randomized for each participant, with the single condition that the NoExo trials always followed each other at either the start or the end of the full experiment. Before starting the Exo block of trials, a familiarization test of $\approx$ 4 min of unperturbed walking was performed in order to let the participants get used to the ankle modules of the Symbitron exoskeleton. Participants could take breaks between trials if needed.

To reduce the effect of the adaptation and anticipation on the results, participants did not receive information about the magnitude or time of the perturbations, nor about if they would be assisted or not. Unavoidably, the participants knew whether a trial was with or without exoskeleton.

### Data acquisition

Ankle joints angles, angular velocities, torques and other data related to the exoskeleton controller were logged at 1000 Hz through the exoskeleton computer. Ground reaction forces and moments were collected by means of the instrumented treadmill at 1000 Hz and used to detect the feet locations, gait phases and trigger the perturbations.

Kinematic data of bony landmarks on the feet, lower legs, upper legs, pelvis and trunk and marker frames on the shanks and thighs, were collected at 128 Hz using a motion capture system with 8 Oqus cameras (Qualisys, Göteborg, Sweden). Markers located on the pelvis were used to estimate the movement of the *COM* in the AP direction, fed back in real-time to the exoskeleton balance controller (Fig. 1). The *COM* position was derived and band-pass filtered (IIR filter, 5–50 Hz, 0.1–40 dB) before being used by the perturbation detection algorithm.

Muscle activity (EMG) of selected muscles around the ankle joints was measured using surface EMG of the tibialis anterior (TA), soleus (SOL), gastrocnemius medialis (GM) and gastrocnemius lateralis (GL). The EMG data were registered bilaterally by means of 8 bipolar electrodes (Bagnoli, Delsys, Natick, MA, USA), sampled at 2048 Hz via Qualisys Track Manager.

All data were synchronized using the ground reaction forces, whose analog signals were logged by both the exoskeleton and Qualisys computers.

### Data processing

Data were processed using Matlab 2018b (Mathworks, Natick, MA, USA). Kinematic data and EMG data were first resampled to 1000 Hz to match the raw data recorded with the exoskeleton.

EMG raw data were pre-processed to obtain linear envelopes removing the effects of noise and artifacts. In particular, we used a bandpass filter (cut-off frequency 30–300 Hz), full-wave rectification, and a low-pass filter (cut-off frequency 3 Hz). All filters were zero-lag 2nd order Butterworth filters. Maximum activations found in the entire 4-min familiarization test (unperturbed walking) with exoskeleton were used to normalize the linear envelopes of each muscle.

For each participant and trial, synchronized and pre-processed data were classified based on the moment of occurrence of the perturbations (DSRF, SSR). Data belonging to the classified perturbations were stored in two different ways: (1) by taking time-intervals of 1 s before and 2.5 s after the perturbation onset; and (2) by normalizing the perturbed gait cycle and the following unperturbed gait cycle (starting each gait cycle with left heel contact). Each gait cycle was linearly interpolated over 100 data points, where single and double support phases were treated separately.

Repetition-averages per walking condition (Exo-No Assistance, Exo-Assistance, NoExo) and perturbation condition (Low and High magnitudes, and DSRF and SSR occurrences) were computed for each participant. Finally, the repetition-average data were averaged across subjects and used to calculate standard deviations (SD).

#### Controller performance

For the Exo-Assistance walking condition, joint angles, angular velocities and exerted torques were used to assess the delivered assistance and its effect on the ankle motion. Moreover, the delay on the detection of perturbations was also registered.

#### Analysis of effort

The effort exerted by the participants to counteract the perturbations was assessed by computing the integrals of the normalized EMG activity of each muscle over 500 ms from the perturbation onset. This time interval of 500 ms was chosen as it captures the main response of the human subjects to counteract the perturbations [14] (Fig. 5).

#### Analysis of *COM* kinematics and spatio-temporal parameters

Measures related to participants’ *COM* kinematics and spatio-temporal parameters were determined after perturbations. The calculated metrics are described below.

The relative maximum *COM* motion in AP direction ($x_{max}$) was assessed after perturbation onset to describe participants’ reactions in terms of peak excursion in the direction of the perturbation. It was calculated with respect to the position of the *COM* at perturbation onset (t = 0 s):

6 $$x_{max} = \max \limits _{t}\left\{ x\left( t\right) - x\left( 0\right) \right\}$$

where *t* is the local time value starting at perturbation onset.

To take into account not only the *COM* position, but also its velocity, we quantified the Recovery Time from $\dot{e}$. This metric evaluates the time needed by the participant to recover from a perturbation (*COM* velocity goes back to its normal range). We considered that the participant recovered if after perturbation onset, $\dot{e}$ remained within ± 0.08 m/s for at least 0.5 s.

We also computed the margin of stability in the AP direction ($MOS_{x}$) [28], which accounts for both the *COM* position and its velocity. This metric varies continuously over the gait cycle, thus, we selected the specific instant of half gait cycle after perturbation onset for its calculation, ensuring that the assistive action, when present, was completed:

7 $$OS_{x} = U_{max} - XcoM$$

where $U_{max}$ is the anterior boundary of the base of support at half gait cycle after perturbation onset (i.e. left toes) and *XcoM* is the extrapolated *COM* in the AP direction computed as:

8 $$XcoM = x + \frac{\dot{x}}{\sqrt{\frac{g}{l_{com}}}}$$

The Step Length after perturbation was estimated as the distance between right and left heels at the instant of next left-heel strike for both, the DSRF and SSR perturbation occurrences.

The gait phases durations within the perturbed gait cycle and the following unperturbed gait cycle were computed. Based on these, the Swing Time to left-heel strike was computed after perturbations.

#### Statistics

Mean and SD were the main descriptive statistics used to summarize the characteristics of data samples. For each defined parameter related to effort, *COM* kinematics and spatio-temporal parameters, we first calculated repetition-averages per participant, which allowed a subsequent average and SD across subjects.

The effects of the Exo-Assistance compared to Exo-NoAssistance walking conditions were assessed by means of a two-way repeated measures ANOVA test with two within factors: ROBOTIC SUPPORT (NoAssistance, Assistance) and PERTURBATION MAGNITUDE (Low, High). Interaction effects of ROBOTIC SUPPORT and PERTURBATION MAGNITUDE combined were also evaluated. The ANOVA was performed on EMG activity, $x_{max}$, Recovery Time, $MOS_x$, Step length and Swing Time.

A Jarque–Bera test determined the normal distribution of the data. A Greenhouse-Geisser correction was applied in case of a lack of sphericity in the data, indicated by Mauchly’s test for sphericity. When interaction effects were present, Tukey’s Honestly Significant Difference was employed as a post-hoc test to compare the support for individual perturbations magnitudes.

A two-sided confidence interval with $\alpha =0.05$ was used to define significance for all statistical tests. All statistical analysis were done using Matlab 2018b.

## Results

The effectiveness of the assistive strategy was assessed in terms of (1) controller performance, (2) analysis of effort and (3) analysis of *COM* kinematics and spatio-temporal parameters. We focused on the comparison Exo-Assistance and Exo-NoAssistance, as in these both walking conditions the participants wore the exoskeleton and thereby, the effect of the controller could be assessed under the same context (i.e. same hardware constrains to the participants’ walking). Nevertheless, NoExo condition was still quantified and served solely as a reference on how participants’ reactions were when they counteracted perturbations without exoskeleton.

The perturbation detection algorithm identified the provided perturbations with an average accuracy of 89.44 ± 5.75% and 95.05 ± 1.78% for Low and High magnitudes respectively. An average of 12 (detected) perturbations were analysed for each participant, walking condition (Exo-NoAssistance, Exo-Assistance, NoExo) and perturbation condition (Low and High magnitudes, and DSRF and SSR occurrences). For participant P3, the SSR perturbations provided during the trial Exo-Assistance-High were excluded from the assessment as they were not properly timed due to an issue with the gait phase detection on this specific case.

### Controller performance

The perturbation detection algorithm was able to identify balance loss in about 200 ms for all conditions (Fig. 2B), moment at which the robotic assistance was triggered for the recovery strategy. Larger perturbation magnitudes were detected faster (detection time generally smaller for High than for Low magnitude), and with higher accuracy (95.05% for High and 89.44% for Low).

**Fig. 3:**
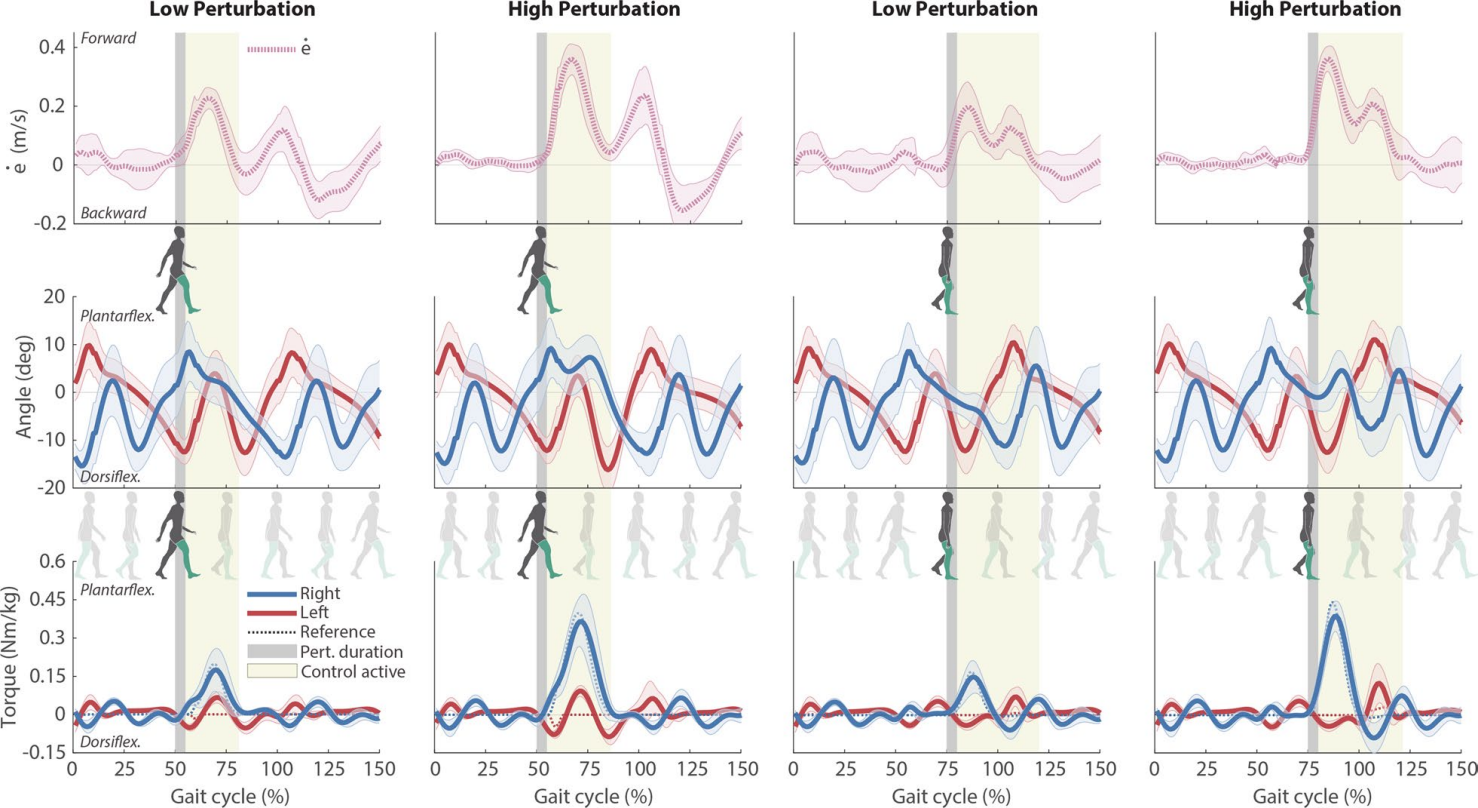
Participants’ kinematic and kinetics data (mean values ± SD) corresponding to the assistance trials and expressed with respect to the normalized gait cycles. *COM* velocity error calculated based on (1) is presented in the first row. Ankle joint angles (second row) and assistive joint torques (third row) are plotted in blue (right ankle) and red lines (left ankle). Reference torques computed by the controller are represented by the dotted lines (the yellow areas indicate the time period in which the controller was active). Vertical dark grey areas represent the perturbation occurrences, and light yellow areas the intervals in which the robotic assistance was being triggered

The balance controller adapted and scaled the robotic assistance based on the perturbation occurrence and perturbation magnitude, according to Eq. (4). The resulting assistive torques are shown in Fig. 3 (third row).

Once the balance was recovered (around 100% gait cycle for DSRF, and 125% gait cycle for SSR, Fig. 3), the adaptive threshold conditions (Eqs. (2) and (3)) were not satisfied anymore, and therefore the reference torques returned to zero again, establishing the transparent mode on the ankle-exoskeleton. Although in transparent mode the generated torques were not perfectly controlled to zero [24], they were close enough (root-mean-square error over a gait cycle of 1.8 Nm) to not interfere with participants’ voluntary movement [24] (see movie on Additional file 1).

The exerted balance assistance torques directly influenced the joint angles (second row in Fig. 3), contributing to participants’ balance recovery by accelerating the *COM* opposite to the perturbation. With DSRF perturbations, the right ankle received plantar-flexion assistance to slow down the forward movement provoked by the perturbation. Meanwhile, the left ankle received dorsi-flexion assistance for the period in which it was in contact with the ground, reducing the toe-off forward propulsion. In case of SSR perturbations, only the right foot initially received assistance after the trigger as the left foot was in swing. In case the participant did not recover before the left foot touched the ground, the plantar-flexion torque that was being applied to the right ankle turned into dorsi-flexion, and the left ankle received plantar-flexion torque as it became the leading foot (see 100–25% gait cycle in SSR torque graphs of Fig. 3).

### Analysis of effort

**Fig. 4:**
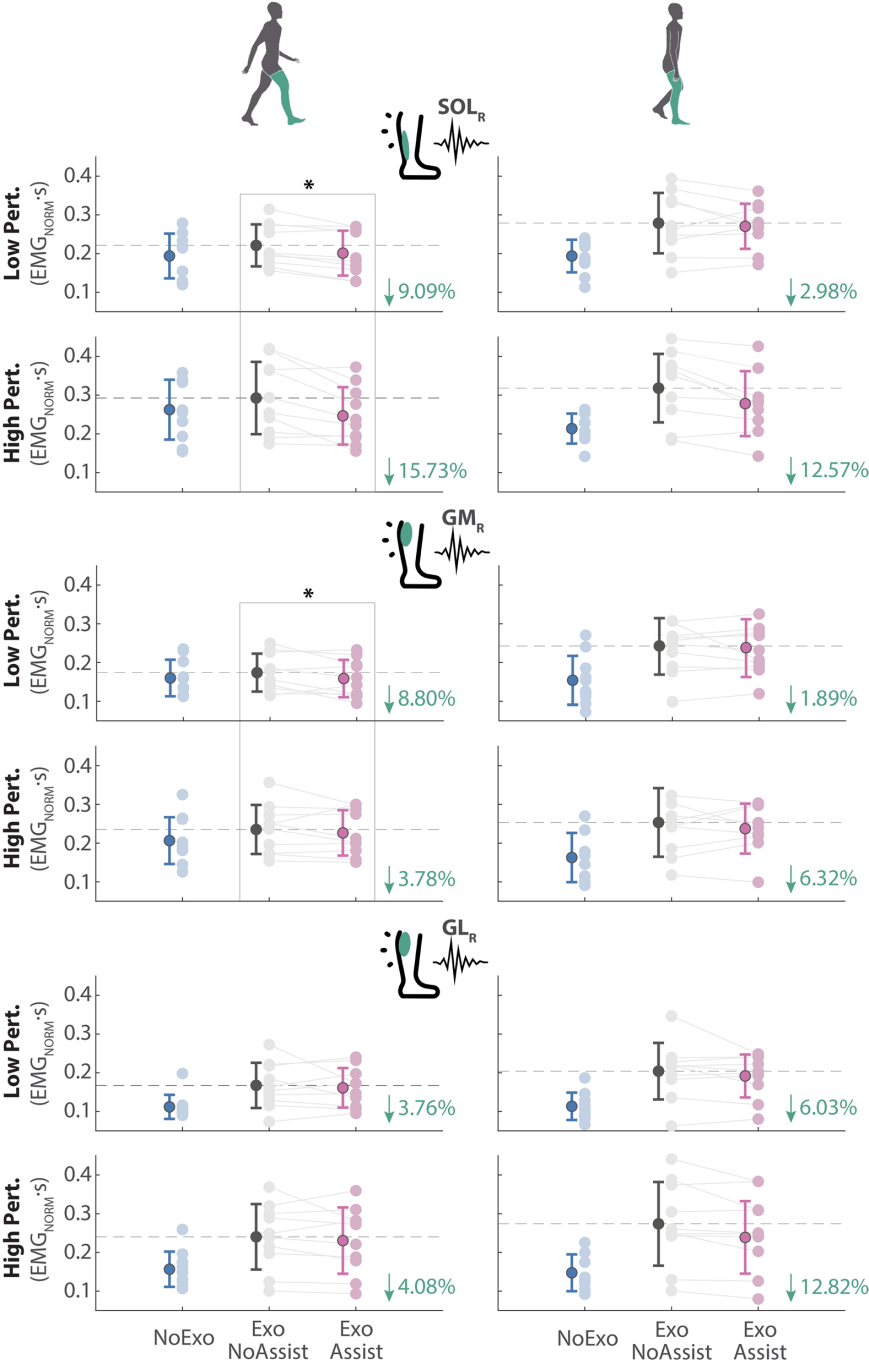
Mean and SD of the integrals of normalized EMG over 500 ms from perturbation onset. Results for SOL$_R$, GM$_R$ and GL$_R$ are represented for DSRF (left column) and SSR (right column) perturbation occurrence, Low and High perturbation magnitudes, and NoExo (blue), Exo-NoAssistance (grey) and Exo-Assistance (pink) walking conditions. Lighter circles stand for individual participant repetition-average, and darker circles stand for mean and SD across participants. The comparison Exo-Assistance vs. Exo-NoAssistance is represented by the percentages and significant differences by (*)

**Fig. 5:**
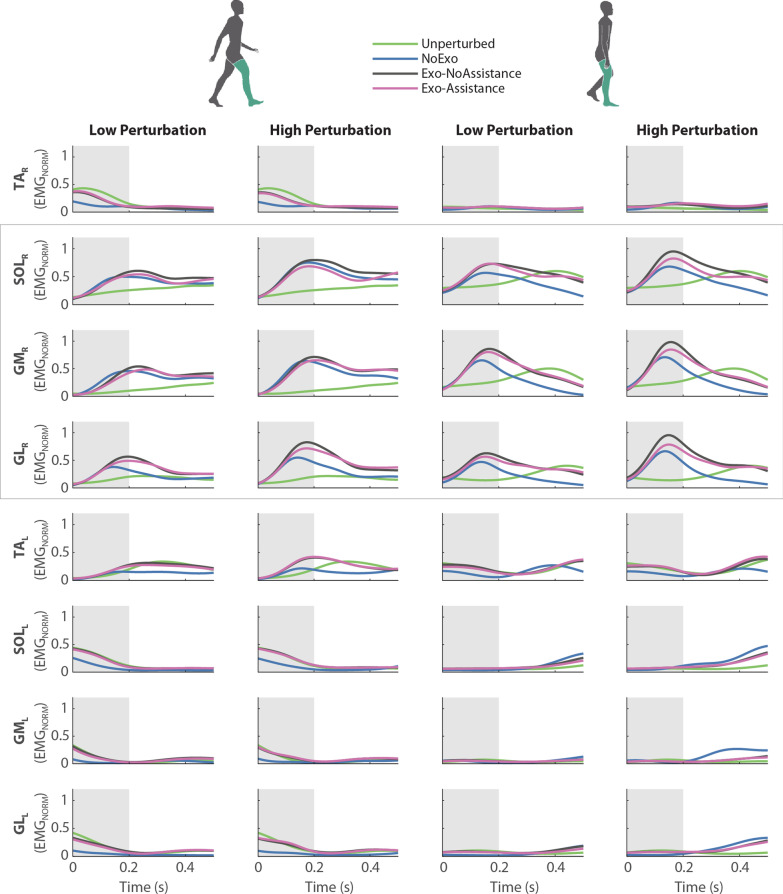
Time-series comparison of normalized muscle activation for each tested walking condition: unperturbed walking (green), user with No Exo (blue), user with Exo-NoAssistance (grey), and user with Exo-Assistance (pink). Each row of graphs corresponds to one muscle. Low and High perturbations for DSRF and SSR are presented in columns. The time interval corresponds to the 500 ms taken to compute the integral for effort assessment. The grey area represent the perturbation duration

Recorded muscles showed clear modulations of EMG activity in response to the perturbations (Fig. 5 in Appendix). These modulations were modified by the application of robotic assistance, generating reductions of EMG activity on the Exo-Assistance trials compared to Exo-NoAssistance trials (Fig. 4 and Table 2). The reductions were especially present for the plantar-flexor muscles of the right leg (SOL$_R$, GM$_R$ and GL$_R$), as they were the main muscles used to counteract the perturbations, and also the primary muscles assisted by the controller (see Fig. 5). For the rest of the recorded muscles (SOL, GM and GL of the left leg, and TA of both right and left) we did not find any notable change or significant difference between Exo-Assistance and Exo-NoAssistance.

**Table 2:** Summary of effort comparison for Exo-Assistance vs Exo-NoAssistance walking conditions

			
SOL$_R$	− 12.41%*	− 7.78%	− 10.09%*
GM$_R$	− 6.29%*	− 4.11%	− 5.20%
GL$_R$	− 3.92%	− 9.42%	− 6.67%

A decrease of effort with Assistance is expressed by the (−) sign. Statistical differences are represented by (*). Compared values correspond to the time-integrals of EMG activity over 500 ms from perturbation onset

The reductions of EMG activity of the specified muscles of the right leg were further analysed for each of the two perturbation occurrences (Fig. 4 and complete statistic analysis in Table 3). Statistical analysis identified that with DSRF perturbations, the EMG activity for the SOL$_R$ was significantly smaller with the Exo-Assistance than with the Exo-NoAssistance (F(1,9) = 17.336, p < 0.01) as well as for the GM$_R$ (F(1,9) = 6.730, p < 0.05). For GL$_R$, although there was a reduction of EMG with Exo-Assistance, no significant differences were found. The effect of the assistance in case of SSR perturbations also resulted in a drop of EMG activity for the mentioned muscles (e.g. 8/9 participants reduced EMG activity for SOL$_R$ with High perturbations), but the statistical tests did not report any significant difference in this case.

**Table 3:** Repeated measures ANOVA tests with two within factors: ROBOTIC SUPPORT (NoAssistance, Assistance) and PERTURBATION MAGNITUDE (Low, High)

		
		F	p	df1	df2	F	p	df1	df2
TA$_R$	PERT. MAGNITUDE	0.662	0.437	1	9	13.844	**0.006**	1	8
	ROBOTIC SUPPORT	2.159	0.176	1	9	0.653	0.446	1	8
	Interaction effects	2.512	0.147	1	9	10.780	**0.011**^†^	1	8
SOL$_R$	PERT. MAGNITUDE	27.457	**< 0.001**	1	9	9.923	**0.014**	1	8
	ROBOTIC SUPPORT	17.336	**0.002**	1	9	1.355	0.278	1	8
	Interaction effects	3.626	0.089	1	9	4.132	0.077	1	8
GM$_R$	PERT. MAGNITUDE	30.249	**< 0.001**	1	9	1.735	0.224	1	8
	ROBOTIC SUPPORT	6.730	**0.029**	1	9	0.027	0.873	1	8
	Interaction effects	0.175	0.686	1	9	0.685	0.432	1	8
GL$_R$	PERT. MAGNITUDE	33.856	**< 0.001**	1	9	17.686	**0.003**	1	8
	ROBOTIC SUPPORT	1.300	0.284	1	9	4.320	0.071	1	8
	Interaction effects	0.031	0.864	1	9	0.023	0.883	1	8
$x_{max}$	PERT. MAGNITUDE	91.973	**< 0.001**	1	9	253.158	**< 0.001**	1	8
	ROBOTIC SUPPORT	5.08e−05	0.994	1	9	2.119	0.184	1	8
	Interaction effects	2.205	0.172	1	9	8.898	**0.017**^‡^	1	8
Recov.time	PERT. MAGNITUDE	12.804	**0.006**	1	9	9.917	**0.016**	1	8
	ROBOTIC SUPPORT	0.093	0.767	1	9	0.077	0.790	1	8
	Interaction effects	0.021	0.887	1	9	2.79e−04	0.987	1	8
$MOS_{x}$	PERT. MAGNITUDE	15.200	**0.004**	1	9	0.054	0.822	1	8
	ROBOTIC SUPPORT	1.111	0.319	1	9	2.817	0.132	1	8
	Interaction effects	0.197	0.668	1	9	1.408	0.269	1	8
Step length	PERT. MAGNITUDE	2.020	0.189	1	9	23.653	**0.001**	1	8
	ROBOTIC SUPPORT	4.210	0.070	1	9	0.045	0.837	1	8
	Interaction effects	6.016	**0.037**^‡^	1	9	2.325	0.166	1	8
Swing time	PERT. MAGNITUDE	38.379	**< 0.001**	1	9	0.300	0.597	1	8
	ROBOTIC SUPPORT	0.679	0.431	1	9	1.744	0.219	1	8
	Interaction effects	3.646	0.089	1	9	2.698	0.135	1	8

*F* and *p* values are reported, as well as the degrees of freedom (df). Bold values represent $p < 0.05$

When interaction effects were present, Tukey’s significant difference was employed as post-hoc test to compare the robotic support for individual perturbation magnitudes. The symbol ^†^ expresses no significance for any of the post-hoc tests, while the symbol ^‡^ represents a significant interaction for the Low perturbation magnitude

The EMG activity was larger with the High perturbation magnitude than with the Low, indicated by a significant effect of the PERTURBATION MAGNITUDE on most of the recorded muscles (Table 3). However, the robotic assistance did not result in more reduction of EMG for High perturbation magnitudes than for Low, and thereby, no interaction effects between ROBOTIC SUPPORT and PERTURBATION MAGNITUDE were found.

The averaged values and SD for the effort (EMG activity) employed by the participants during NoExo walking condition were added for a visual comparison (blue data in Fig. 4). Appendix shows that the triggered EMG responses with or without exoskeleton had a similar shape, but merely with a larger amplitude during Exo trials. However, the weight of the exoskeleton and the constrains that it might introduce to participants’ normal walking, make a direct assessment of difference in responses between NoExo vs. Exo walking conditions not meaningful.

### Analysis of *COM* kinematics and spatio-temporal parameters

In general, none of the defined metrics for *COM* kinematics ($x_{max}$, Recovery Time and $MOS_x$) and spatio-temporal parameters (Step Length and Swing Time) showed a significant influence of the Exo-Assistance comparing with Exo-NoAssistance (see statistics reported in Table 3). This gives an indication that participants’ stability reaction was not significantly affected by the robotic support received even with the exposed reduction of effort. Although the magnitude of the perturbation had an effect on participants’ response, no significant interaction effects between ROBOTIC SUPPORT and PERTURBATION MAGNITUDE were found for any of the metrics, except for the case of $x_{max}$ with Low-SSR perturbations and Step Length with Low-DSRF (Table 3).

## Discussion

### Summary of the results

The present study was designed to determine the effects of a novel ankle-exoskeleton controller in assisting and cooperating with able-bodied participants in balance recovery during walking. Our findings indicate that the developed controller can detect the onset of an unexpected disturbance and effectively trigger a mitigation strategy, which assists the user by requiring less human effort to perform the recovery. In this study, the performance of the controller was tested when forward perturbations were provided. Still, in essence, the proposed controller should also be able to assist users with the required support to counteract backward perturbations (see Eq. (4)), but further testing is required.

Specifically, our perturbation detection algorithm based on $\dot{x}$ real-time feedback was able to identify the loss of balance in less than 200 ms (Fig. 2B). This duration is certainly small compared with previous literature (350 ms in Monaco et al. [15]), and has the potential to be used as a method for falling avoidance in the future [29].

Our approach does not require any complex subject-tailoring procedure (beyond the information of subject’s mass and pelvis height) for successful application among different subjects. The perturbation detection algorithm is based on the comparison between actual and predicted *COM* kinematics (Fig. 1). As the adaptive threshold is adjusted based on preceding gait cycles, once the subjects had performed a certain number of unperturbed steps at a constant speed, the algorithm was able to identify *COM* disturbances without requiring further subject-specific training. However, a possible shortcoming of this detection method is its limitation to a constant walking speed. We hypothesise that if a subject would continually change speeds during walking, the adaptive threshold would get less sensitive for the detection of perturbations. To confirm this hypothesis, further testing (more variable conditions and populations) of the perturbation detection algorithm is required, which was out of the goal of this paper.

By using the perturbation detection algorithm, our controller assisted the balance of the user only if required, and adapted the robotic support to the provided perturbation and user’s reaction within the gait cycle. The assistance was easily normalized to the perturbation magnitude and user’s characteristics by taking into account the $\dot{e}$ signal, subject’s mass and pelvis height (Eq. (4)). Moreover, the assistance also modulated with the vertical ground reaction force *F*, which allowed a smoother distribution of assistive torques across legs and a more natural kinematic behaviour [17].

In the present study, it was hypothesized that when wearing the exoskeleton, the developed balance controller would reduce the human contribution to the recovery response without negatively impacting their stability. This was indeed the case, as the effect of the torque-based assistance on the ankle joints resulted in a reduction of the EMG activity of the primary muscles contributing to the balance recovery response. Concretely, average reductions of soleus activity (10.09%) and gastrocnemius medialis activity (5.20%) were observed in the stance leg (Table 2), including both DSRF and SSR perturbations. These observed reductions only reached significance when perturbations were applied at DSRF (not for SSR). A possible explanation might be that as perturbations applied at SSR occurred later during the stance phase, there was less effective time to induce an effect that would lead to reductions in EMG.

To the best of our knowledge, no outcomes on EMG activity have been earlier reported in literature specifically covering ankle-exoskeleton balance controllers during walking. On one hand, Jackson and Collins [21] previously showed that a co-adaptive algorithm could led to ankle-exoskeleton assistance that reduced soleus activity during unperturbed locomotion. Here we go further and demonstrate that the ankle-exoskeleton assistance can also adapt the modulation to unexpected perturbations, cooperating with the users and decreasing their effort. On the other hand, in [19], authors also reported a decrease of soleus activity when providing the ankle assistance during standing balance. In this case, we have extended the positive results also to the walking condition. Contrarily to the two above-mentioned studies, we did find reductions of gastrocnemious medialis and lateralis activity apart of the reported soleus activity.

Despite the reduction of human effort, we did not find significant changes on participants’ *COM* kinematics and spatio-temporal parameters. The reason why there was not a clear improvement of these metrics might be explained by the fact that all participants were able-bodied subjects, and thereby, there was no need for further balance improvement. Humans may prefer to reduce effort when they are confidently able to maintain balance [19], and consequently rely on the assistance received, encouraging the “slacking effect” [30]. This effect can be the reason why when receiving assistance, participants’ stability was kept equally good, having a preference for the reduction of EMG activity.

### Limitations and future directions

The balance controller presented in this study is mainly focused on the ankle joint, as it was demonstrated to be a primary stabilization action done by the stance leg for perturbations occurring on the sagittal plane [13, 14, 18]. Although the sagittal is the primary plane during human locomotion, human balance control is a three-dimensional problem, and foot placement adjustments in both the sagittal and frontal planes might be required. However, a control of the ankle joint in the frontal plane (i.e. ankle inversion/eversion), would only allow for limited improvement in medio-lateral direction. As previously presented in the literature [15–17], such foot placement adjustments can be better achieved by hip strategies on the swing leg. For future studies, the complementary strategies (stance leg stabilization and foot placement adjustments) should be combined on an improved hip + ankle controller, which we believe will enrich the balance controller performance and will better cooperate with users in recovering stability. In this direction, the effect of the complementary strategies (hip + ankle) should be assessed not only with forward but also with backward perturbations.

Moreover, ankle-exoskeletons are becoming more and more regarded and relevant to improve mobility and stability of people with motor impairments [9, 19]. A future consideration to be investigated is the potential application of this controller to improve walking balance of people with such walking limitations. One fundamental advantage of our approach is its cooperative behaviour, which allowed “assist-when-needed” the able-bodied participants of this study. It is unknown how patients of diverse pathologies (e.g. spinal cord injury, cerebral palsy or stroke) would respond to the provided assistive torques while having difficulties to collaborate themselves. Therefore, a future direction should be focus on testing the ankle-exoskeleton controller on such patient groups.

Finally, to be able to use the proposed controller as a solution outside of the lab environment, it would be necessary to get reliable *COM* state and vertical ground reaction forces information independent of a fixed motion capture system. Two feasible measurement strategies to achieve this might be: (1) to estimate the whole body *COM* from a single inertial measurement unit (IMU) during walking [31], and/or (2) to estimate the whole body *COM* state and ground reaction forces from three IMUs during walking [32, 33].

## Conclusion

In this study we investigated the effects of a cooperative ankle-exoskeleton controller on the dynamic balance of able-bodied subjects receiving external perturbations during walking. The presented approach has the ability to tailor the assistance to the user’s characteristics and responses, distributing the required support over both legs depending on the phase of walking.

Overall, our findings show the potential of the cooperative ankle controller to assist humans’ balance only if required, reducing user’s effort. The results obtained with able-bodied participants hold promise for using the controller on an exoskeleton to assist people with motor disabilities in improving balance control. In future studies, it will be required to assess the effect of the controller also when backward perturbations are provided.

### Supplementary Information

**Additional file 1.** Video material showing the experiment carried out and the results presented in the manuscript.

## Data Availability

The datasets generated and/or analyzed during the current study are available from the corresponding author on reasonable request.

## Appendix

Here we report the time-series averages for normalized EMG data for all recorded muscles (Fig. 5): tibialis anterior (TA), soleus (SOL), gastrocnemius medialis (GM) and gastrocnemius lateralis (GL) of right (R) and left (L) leg.

The use of the exoskeleton (grey and pink lines in Fig. 5) triggers the same shape of EMG responses than when the participants were perturbed without exoskeleton (blue line in Fig. 5), but just with a larger amplitude due to the exoskeleton weight.

Signals corresponding to perturbed trials start differing from the unperturbed trial (green line in Fig. 5) after perturbation onset.

## Abbreviations

*COM*: Center of mass
*XcoM*: Extrapolated center of mass
*AP*: Antero-posterior
*x*: Center of mass position in AP direction
$\dot{x}$: Center of mass velocity in AP direction
$\hat{\dot{x}}$: Predicted center of mass velocity in AP direction
$\dot{e}$: Center of mass velocity error
$$\tau$$: Robotic torque
*F*: Vertical ground reaction force
*M*: Subject’s mass
$l_{com}$: Subject’s *COM* height
DSRF: Start of the double support phase with right foot leading
SSR: Mid single stance right (left foot in swing)
EMG: Electromyography
TA: Tibialis anterior
SOL: Soleus
GM: Gastrocnemius medialis
GL: Gastrocnemius lateralis
SD: Standard deviation
IMU: Inertial measurement unit
